# Effect of Rhizobium Symbiosis on Low-Temperature Tolerance and Antioxidant Response in Alfalfa (*Medicago sativa* L.)

**DOI:** 10.3389/fpls.2019.00538

**Published:** 2019-04-30

**Authors:** Yu-Shi Liu, Jin-Cai Geng, Xu-Yang Sha, Yi-Xin Zhao, Tian-Ming Hu, Pei-Zhi Yang

**Affiliations:** ^1^Department of Grassland Science, College of Animal Science and Technology, Northwest A&F University, Yangling, China; ^2^Shaanxi Grassland Workstation, Xi’an, China

**Keywords:** alfalfa, low-temperature tolerance, antioxidant response, rhizobium symbiosis, low temperature regulated genes

## Abstract

Low temperature-induced stress is a major environmental factor limiting the growth and development of plants. Alfalfa (*Medicago sativa* L.) is a legume well known for its tolerance of extreme environments. In this study, we sought to experimentally investigate the role of rhizobium symbiosis in alfalfa’s performance under a low-temperature stress condition. To do this, alfalfa “Ladak^+^” plants carrying active nodules (AN), inactive nodules (IN), or no nodules (NN) were exposed to an imposed low temperature stress and their survivorship calculated. The antioxidant defense responses, the accumulation of osmotic regulation substances, the cell membrane damage, and the expression of low temperature stress-related genes were determined in both the roots and the shoots of alfalfa plants. We found that more plants with AN survived than those with IN or NN under the same low temperature-stress condition. Greater activity of oxidation protective enzymes was observed in the AN and IN groups, conferring higher tolerance to low temperature in these plants. In addition, rhizobia nodulation also enhanced alfalfa’s ability to tolerate low temperature by altering the expression of regulatory and metabolism-associated genes, which resulted in the accumulation of soluble proteins and sugars in the nodulated plants. Taken together, the findings of this study indicate that rhizobium inoculation offers a practical way to promote the persistence and growth potential of alfalfa “Ladak^+^” in cold areas.

## Introduction

Alfalfa (*Medicago sativa* L.) is a widely cultivated forage crop of substantial economic value that possesses excellent agricultural traits, in that its roots can fix atmospheric nitrogen molecules with the help of symbiotic rhizobia (Hou et al., 2013; Quan et al., 2016; Zhang W. et al., 2017). In turn, the endogenous nitrogen pool accumulated in the root system may enhance the cold-tolerance ability of this plant (Dhont et al., 2006). Yet, little research has actually investigated the effect of rhizobium symbiosis on alfalfa’s tolerance of low temperatures. Earlier work demonstrated that inoculated rhizobia improved the productivity and survival of legumes under low temperature conditions (Prévost et al., 1999, 2003). And elevated CO_2_ has been shown to stimulate rhizobium-inoculation alfalfa growth and to reduce this plant’s freezing tolerance (Bertrand et al., 2007).

Low temperatures adversely affect crop survival, growth, and productivity (Dimkpa et al., 2009; Chinnusamy et al., 2010), typically prompting various physiological and biochemical changes in plants, including alterations to membrane permeability and enzyme activities (Hu et al., 2016). A low temperature elicits the generation of reactive oxygen species (ROS), such as hydrogen peroxide (H_2_O_2_), superoxide radical (O_2_^∙-^), and hydroxyl radical (OH^∙^), causing severe oxidative stress. Oxidative damage not only results in the oxidation of cellular components, which leads to protein dysfunction and DNA damage, but it also harms the cell membrane’s lipids. As a key negative product in the signaling network of plants’ stress responses, ROS can disrupt the plant membrane structure then generate malondialdehyde malondialdehyde (MDA), a by-product that is highly reactive and able to cause secondary oxidative damage (Gill and Tuteja, 2010; Erdal et al., 2015; Chen et al., 2016; Jin et al., 2016).

To alleviate this oxidative damage, plants have evolved protective enzymatic defense systems to detoxify ROS and to reduce oxidative stress, such as those relying on peroxidase (POD, EC 1.11.1.7), superoxide dismutase (SOD, EC 1.15.1.1), and catalase (CAT, EC 1.11.1.6). These traditional antioxidant enzymes work together to detoxify ROS, but mounting evidence also suggests proline can function as a non-enzymatic antioxidant by regulating osmosis and detoxifying ROS (Trovato et al., 2008). Moreover, the accumulation of osmotic adjustment substances, namely soluble proteins and sugars, can further contribute to the tolerance of low temperature-induced stress in plants (Castonguay et al., 1995).

Under stressful environmental conditions, crop plants activate their stress-responsive genes involved in ROS homeostasis regulation. Examples include *ProDH*, encoding proline dehydrogenase, being downregulated when a plant experiences drought or low temperature (Xin and Browse, 1998); the *CorF* gene, encoding galactinol synthase, which regulates osmosis and maintains membrane integrity by controlling the synthesis of soluble sugars (Liu et al., 2016); Cas, a member of the dehydrin protein family, produced in response to cold or drought stress (Monroy et al., 1993; Wolfraim and Dhindsa, 1993); and *CBF2*, a transcription factor related to the low- temperature tolerance of plants (Thomashow, 2010; Shu et al., 2017).

Because of its low level of autumn dormancy, the alfalfa “Ladak^+^” cultivar is often planted in northwestern China, especially in the deserts of Xinjiang. But the air temperatures in these deserts varies greatly from day to night, often reaching highs of 35°C and lows of -6°C; this clearly imposes a stress upon these crop plants. We hypothesized that rhizobium symbiosis may improve the low-temperature tolerance of alfalfa “Ladak^+^” by affecting its physiological and biochemical processes. To test this, we evaluated the tolerance to cold of alfalfa “Ladak^+^” plants with active nodules (AN), inactive nodules (IN), and no nodules (NN), by comparing their respective survival and electrical conductivity under 0 and -6°C, activity of antioxidative enzymes, alterations in osmolyte adjustment, and the expression profiles of low temperature-related genes at 0°C.

## Materials and Methods

### Plants, Their Growing Conditions, and Nodulation Treatments

The seeds of alfalfa (*Medicago sativa*) (Ladak^+^, United States) were first rinsed with 70% ethanol for 30 s, then with a 0.5%-NaClO solution for 15 min, and finally thrice with sterile water. All seeds were germinated on wet filter paper in Petri dishes for 5 days in a plant growth chamber at 25°C and 70% relative humidity, with a 16 h photoperiod.

Six days old seedlings were then individually transplanted into plastic conical pots containing sterilized silica sand (100 mesh), followed by sterilization with a 0.5% NaClO solution and three-times rinsed with running water. The seedlings were cultivated under a normal day (30 ± 5°C) and night (20 ± 5°C) cycle, with a relative humidity that ranged between 55 ± 5% and 70 ± 5%, in the greenhouse of Grassland Science Department of Northwest A&F University.

When the seedlings reached a height of 10 cm, they were randomly divided into three groups: (I) AN: alfalfa plants inoculated with the *Rhizobium meliloti* strain Dormal to form AN, supplemented with 1/4 strength nitrogen-free Hoagland solution (Hoagland and Arnon, 1950) daily; (II) IN: alfalfa inoculated with the same *Rhizobium meliloti* strain Dormal as the AN group, but watered daily with 1/4 strength Hoagland solution (Wang et al., 2016); (III) NN: alfalfa without rhizobia inoculation, watered daily with 1/4 strength Hoagland nutrient solution.

**FIGURE 1:**
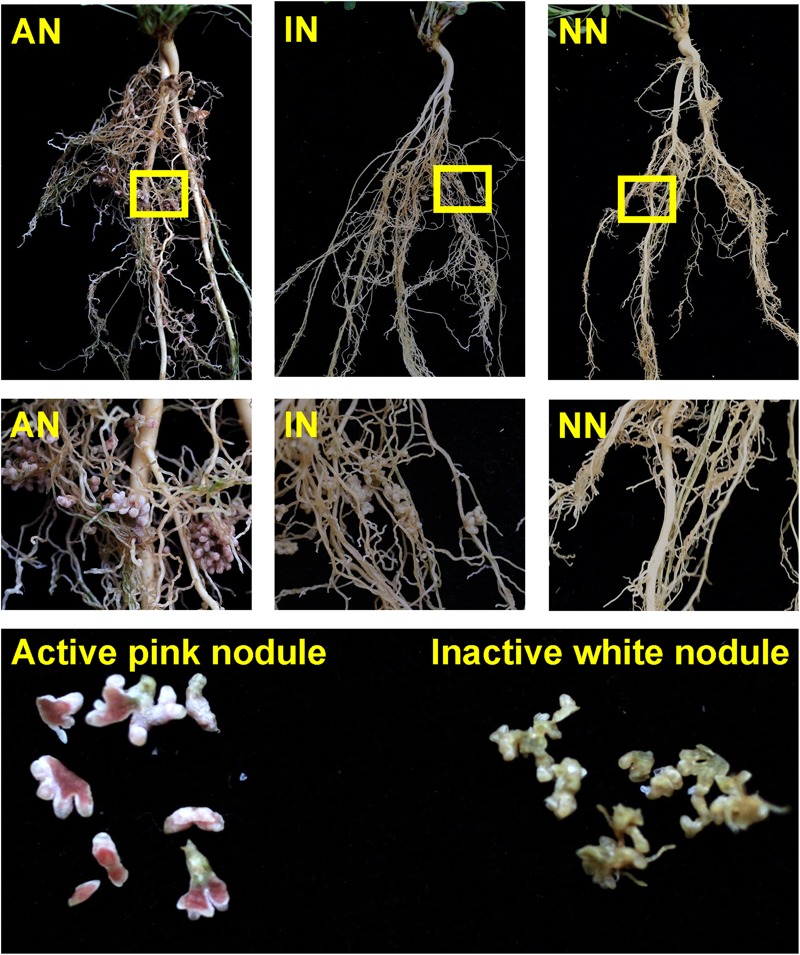
Roots of AN, IN, and NN groups. (AN, alfalfa with active nodules; IN, alfalfa with inactive nodules; NN, alfalfa with no nodules.) Yellow arrows show the pink nodules in the roots of the AN group and the white nodules in the roots of the IN group.

Plant shoots of all three groups were cut at the base of the stem and their biomass weighed on days 60 and 90 of the experiment. After this shoot removal and subsequent plant regrowth, the intended root nodule inoculation treatments were achieved at 120 days: pink nodules were present in the AN roots, white nodules were observed in the IN roots, and NN were observed in the NN roots (Figure 1). These 120 days old seedlings were then subjected to the low temperature treatments, as described below.

### Low Temperature Treatments

The 120 days old alfalfa seedlings of AN, IN, and NN groups were exposed to low temperature treatments of 0 and –6°C in a reconstructed refrigerator. To ensure whole plants (shoot and root parts) were treated at the same given temperature, a temperature controller reduced the ambient air temperature in the refrigerator at a rate at –2.5°C per hour. Hence it took 10 or 16 h to reach 0 or –6°C, respectively, at which plants remained in their targeted temperatures for another 8 h. For their physiological index determinations, the shoots and roots of plants were harvested separately at 0, 2, 4, 6, and 8 h since imposing the low temperature stress treatment. The samples were washed with distilled low-temperature water to remove any sand, then dried with paper towels, and immediately frozen in liquid nitrogen for storage at –80°C.

### Survivorship

At each sampling point in time, 50 treated plants per group were removed at random from the refrigerator. The temperature of plants was restored to room temperature at a rate of +2.5°C per hour, after which the plants were irrigated with their respective nutrient solutions for another 2 weeks. During the recovery period, those plants which had maintained or regained green coloring on their ground parts, or developed new green shoots, were considered to have survived the low temperature stress treatments.

### Biochemical Analyses

Fresh alfalfa leaves were used for electrical conductivity testing, whereas frozen samples were used for all other biochemical assays. All spectrophotometric analyses were conducted on a HITACHI spectrophotometer (UV-3900, Japan). Electrical conductivity was determined as previously described by Song et al. (2006) with minor modifications. Briefly, fresh alfalfa leaves after low temperature stress were gathered and soaked in distilled water for 2 h at 4°C; next, the conductivity value was read as L1 by a conductivity meter DDS-307 (Leici Corporation, China). The mixture was then heated in a boiling water bath for 20 min and the conductivity value L2 was collected once it had cooled down to room temperature. Relative electrical conductivity was calculated as (L1/L2) × 100%. Malondialdehyde (MDA) was measured by using the thiobarbituric acid (TBA) reaction (Guo et al., 2010). Briefly, about 0.5 g of alfalfa tissue was homogenized in 5 ml of a cooled potassium phosphate buffer (pH = 7.8). After centrifuging at 4000 × *g* for 10 min, 2 ml of supernatant was mixed with 2 ml of 0.6% TBA acid and then incubated in a boiling water bath for 20 min. This mixture was chilled rapidly and then centrifuged again at 4000 × *g* for 10 min to remove debris. The absorbances at 532, 600, and 450 nm were measured on a spectrophotometer.

Peroxidase activity (POD) was determined using a guaiacol (C_7_H_8_O_2_) substrate, as described in Xu et al. (2011). Frozen alfalfa plant tissue (approximately 0.2 g) was homogenized in 10 ml of a 50 mM potassium phosphate buffer containing 1% polyvinylpyrrolidone and 1 mM EDTA. The homogenate was centrifuged at 15,000 × *g* for 15 min at 4°C. The supernatant was used to quantify POD activity by measuring the oxidation of guaiacol; the reaction mixture contained 8 mM C_7_H_8_O_2_, 50 mM potassium phosphate buffer, and 2.75 mM H_2_O_2_, with the increase of absorbance at 470 nm measured.

Superoxide dismutase activity (SOD) was determined by using nitroblue tetrazolium (NBT), as described by Giannopolitis and Ries (1977). Specifically, SOD is measured by the reaction mixture’s ability to inhibit the photochemical reduction of NBT. The plant material was homogenized in a 50 mM phosphate buffer (pH 7.8) containing 100 μM EDTA, and 1% (w/v) polyvinyl pyrrolidone (PVP-40), on an ice bath. The homogenate was centrifuged at 12,000 × *g* for 15 min and the ensuing supernatant transferred to a mixture containing 50 mM of phosphate buffer, 130 mM of methionine, 750 μM of NBT, and 20 μM of riboflavin (pH 7.8). The absorbance at 560 nm was monitored.

Catalase activity (CAT) was determined as earlier described by Chance and Maehly (1955). This approach used a CAT reaction solution that consisted of 100 mM phosphate buffer (pH 7.0) and 100 mM H_2_O_2_. The consumption of H_2_O_2_ was then inferred by the decrease in optical density recorded at 240 nm.

Proline content determination followed the acidic ninhydrin reagent method (Bates et al., 1973). Approximately 0.5 g of alfalfa tissue material was homogenized in 10 ml of 3% aqueous sulfosalicylic acid, and then centrifuged at 1000 × *g*. Two ml of the supernatant was reacted with 2 ml of ninhydrin reagent and 2 ml of glacial acetic on a boiling water bath for 1 h. The reaction mixture was extracted with 4 ml of toluene, after which the absorbance of the supernatant was read at 520 nm.

Soluble protein concentration was measured according to the method of Bradford (1976). Specifically, approximately 0.5 g of alfalfa plant tissue was homogenized and centrifuged at 6000 × *g* and 4°C in a phosphate buffer (pH = 7.8). Then the supernatant was mixed with the Bradford reagent, and the absorbance at 595 nm was measured. Soluble sugar content was assayed by using the anthrone reagent (Dreywood, 1946). The alfalfa plant tissue was first mixed with absolute ethanol, and this mixture heated at 80°C for 0.5 h and then centrifuged at 4000 × *g* for 10 min. Two ml of the supernatant was mixed with 5 ml of anthrone reagent-sulfuric acid and incubated on a boiling water bath for 10 min. Finally, the absorbance at 625 nm was measured.

### Plant RNA Extraction, Reverse Transcription, and qRT-PCR Analysis

Total RNA was extracted from the shoots and roots of alfalfa plants exposed to the low temperature treatment (0°C) for 0, 2, 4, 6, and 8 h, by using the Eastep total RNA extraction kit (Promega, China). First strand cDNA was synthesized with the SuperScript II reverse transcriptase (Invitrogen, United States) and the qRT-PCR was performed using the primers listed in Supplementary Table S1 The *β-Actin* gene was used as an internal reference (Zhang et al., 2016), and relative expression levels were calculated using the standard 2^-ΔΔCt^ algorithm (Livak and Schmittgen, 2001). The qRT-PCR was carried out on the Roche LightCycler 4800II Real-time PCR system, with a SYBR Green-based PCR assay used (ABM EvaGreen 2 × qPCR MasterMix–No Dye). Every qRT-PCR sample contained 2 μl of cDNA, 10 μl of SYBR Green, and 2 μl of primer. The qRT-PCR cycle parameters were as follows: 10 min at 95°C, and then 40 cycles of 15 s at 95°C and 1 min at 60°C.

### Statistical Analyses

The experiment consisted of three nodulation treatments (AN, NN, and IN), two low temperature treatments (0 and -6°C), and five sampling time points (0, 2, 4, 6, and 8 h of low temperature stress). One-way ANOVAs were used to determine whether there was a significant difference among treatment means (ANOVA tables are attached in the Supplementary Material), with pairwise mean differences compared by the least significant difference test (LSD) at an alpha level of 0.05. In this study, 54 plants (3 nodulation treatments × 3 biological replicates × 6 individuals) were used for the biomass determination. For the survival determination, we set two temperature conditions (0 and -6°C). And under each temperature, 2250 plants (3 nodulation treatments × 3 biological replicates × 5 sampling times × 50 individuals) were used. To measure the relative electric conductivities under the two low temperatures, for each 135 plants were used (3 nodulation treatments × 3 biological replicates × 5 sampling times × 3 individuals). However, only those plants treated at 0°C were selected to analyze the other plant physiological indices. Finally, 135 plants (3 nodulation treatments × 3 biological replicates × 5 sampling times × 3 individuals) were used for the determination of expression profiles of low temperature-related genes under 0°C. All data were analyzed using SPSS v19.0 software (SPSS IBM, United States) and the figures drawn in GraphPad Prism 5 (San Diego, CA, United States).

## Results

### Rhizobium Nodules Enhanced the Low-Temperature Tolerance of Alfalfa

**FIGURE 2:**
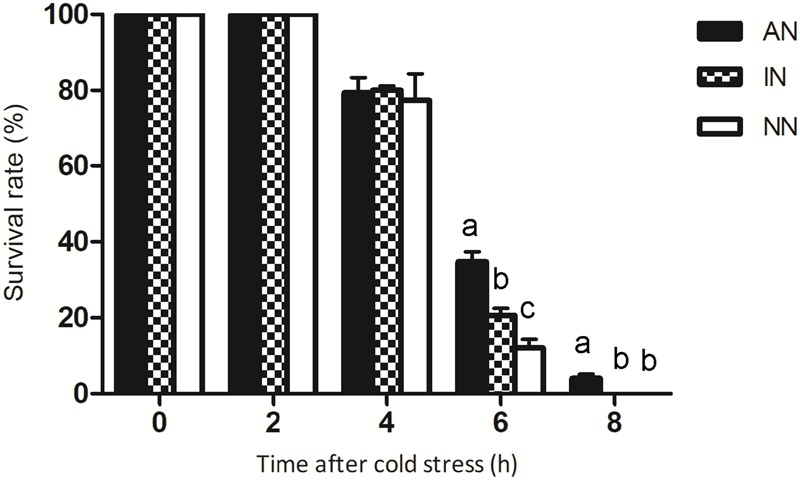
Survival of alfalfa plants under –6°C low temperature stress. The data are means ± SE, *n* = 3. Different letters indicate a significant difference between means (*P* < 0.05).

To confirm that plants were acquiring the same level of nitrogen within a treatment, we measured their harvested aboveground biomass at days 60, 90, and 120 of the experiment. No significant difference in aboveground biomass was observed among the three groups alfalfa (Supplementary Figure S2). All three groups had 100% survival at 0°C, but after 4 h of exposure to –6°C the alfalfa seedlings’ survival was significantly reduced (Figure 2). After 6 h of exposure, survival of the AN and IN groups was significantly higher than that of the NN group, with more AN than IN plants surviving the low temperature stress. After 8 h exposure, only a portion of the AN group had survived, whereas this long-term low temperature stress killed all plants of both IN and NN groups.

### Effect of Rhizobium Symbiosis on Cell Membrane Damage in Alfalfa

Throughout the 8 h exposure of plants to a 0°C-low temperature, the relative electrical conductivity of AN, IN, and NN groups did not significantly increase (Supplementary Figure S3). This indicated that the damage to cell integrity caused by low temperature stress at 0°C was insufficient to cause leakage of intercellular fluid. Comparatively, under –6°C, the electrical conductivity of AN, IN, and NN groups were significantly elevated over time (Supplementary Figure S3), yet no significant difference was observed them at all time points within this temperature treatment. More importantly, the observed higher relative electrical conductivity under –6°C indicated a greater level of internal disorder, and it exceeded the determination limits (Supplementary Figure S3). Therefore, the detection of other physiological indicators was only tested in the 0°C-treated plants.

**FIGURE 3:**
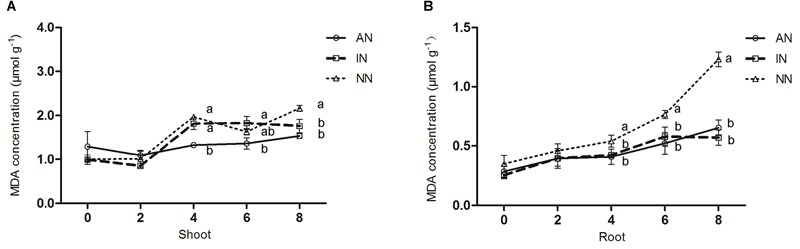
Effect of nodulation on MDA contents **(A,B)** in alfalfa shoots and roots under 0°C. The data are means ± SE, *n* = 3. Different letters indicate a significant difference between means (*P* < 0.05).

Under 0°C, the MDA concentration in the shoots of all three groups of alfalfa were increased (Figure 3A), but the AN group apparently impeded the generation of MDA in its shoots more efficiently than those of NN. In the roots, the concentrations of MDA in all plants were elevated by the low temperature stress at 0°C (Figure 3B). However, the MDA concentration in the NN group significantly exceeded that in the AN and IN groups by 1.90 times and 2.16 times, respectively.

### Effect of Rhizobium Symbiosis on Alfalfa’s Antioxidant Defenses

**FIGURE 4:**
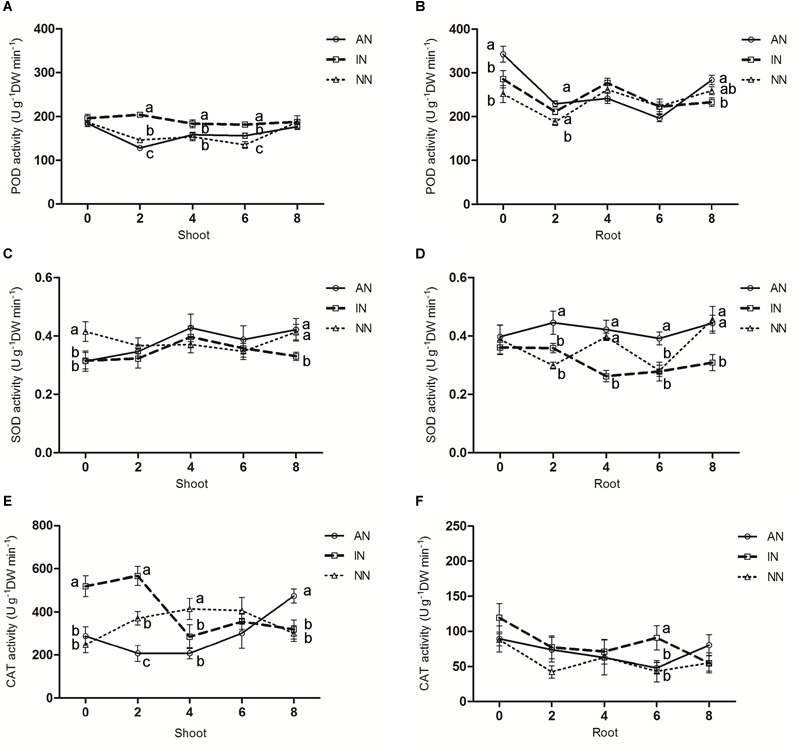
Effect of nodulation on the activities of POD **(A,B)**, SOD **(C,D)**, and CAT **(E,F)** in alfalfa shoots and roots under 0°C. The data are means ± SE, *n* = 3. Different letters indicate a significant difference between means (*P* < 0.05).

POD activity in rhizobium-treated or non-treated alfalfa shoots did not show the same patterns under 0°C. The IN group had significantly higher POD activity than AN or NN at 2 and 6 h of exposure duration (Figure 4A), however, all three groups’ POD activities were similar at 8 h. In the root parts, POD activity of the AN group was significantly higher than those of NN or IN at 0, 2, and 8 h (Figure 4B). As the low temperature treatment continued, the SOD activity in the shoots of the AN and NN groups were maintained at a constant high level (Figure 4C). A significantly higher SOD activity in AN group’s roots than those of NN or IN groups (Figure 4D). In the shoots, CAT activity in the AN group gradually increased under 0°C, whereas initially it increased faster in the NN group but decreased under prolonged incubation, leaving AN with and the highest CAT activity after 8 h. The IN group had the highest CAT activity at 0 h but this declined rapidly soon afterward, so that it was on par with NN group at 8 h (Figure 4E). By contrast, in the roots, no significant difference in CAT activity was observed among the AN, IN, and NN groups after 8 h (Figure 4F).

### Effect of Rhizobium Symbiosis on Soluble Substances in Alfalfa

**FIGURE 5:**
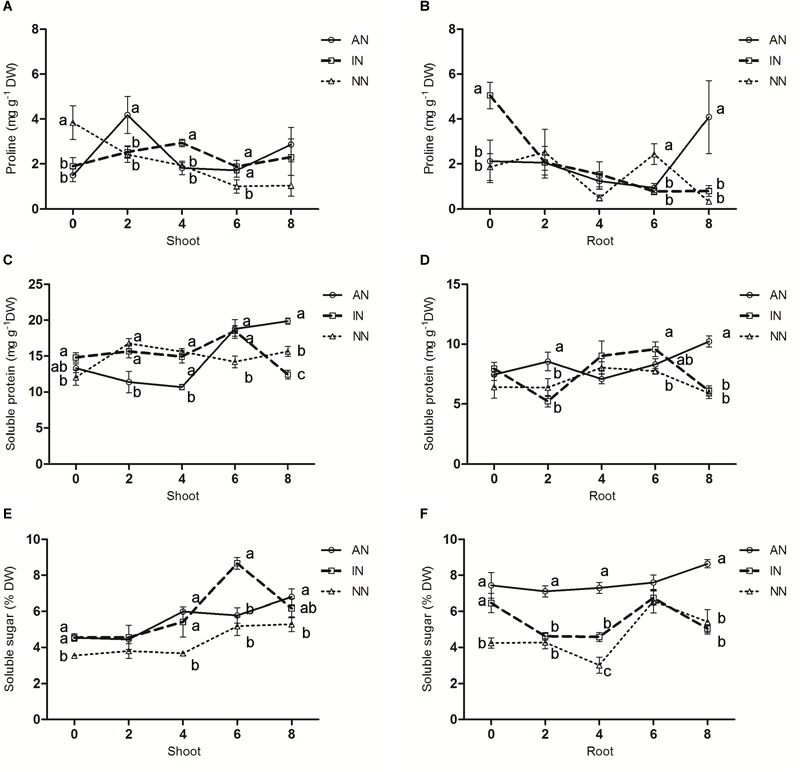
Effect of nodulation on the contents of proline **(A,B)**, soluble protein **(C,D)**, and soluble sugar **(E,F)** in alfalfa shoots and roots under 0°C. The data are means ± SE, *n* = 3. Different letters indicate a significant difference between means (*P* < 0.05).

The proline contents of shoots in the NN group continually decreased under a low temperature treatment of 0°C (Figure 5A), whereas, rhizobium-inoculated alfalfa were capable of sustaining a constant proline level in their shoots in response to the low temperatures. In their roots, the AN group accumulated proline through 8 h of low temperature exposure (Figure 5B). As shown in Figure 5C,D, AN in the AN group assisted protein accumulation in the host plant cells, resulting in a greater level of soluble proteins in both shoots and roots compared with the NN and IN groups. Under prolonged low temperature stress at 0°C, the soluble sugar contents of shoots in the three nodulation groups presented similar increasing trends (Figure 5E). However, compared with NN and IN, the roots of the AN group accumulated more soluble sugar during the whole 8 h period (Figure 5F).

### Effect of Rhizobium Symbiosis on Low Temperature-Regulated Genes

**FIGURE 6:**
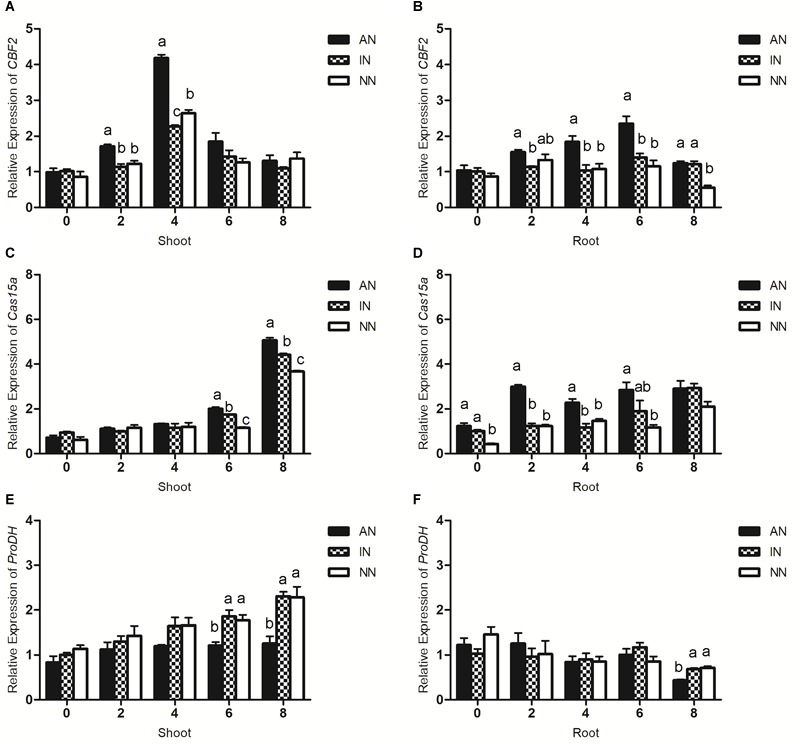
Effect of nodulation on the response of *CBF2* **(A,B)**, *Cas15a* **(C,D)**, and *ProDH* **(E,F)** genes to low temperature stress in alfalfa shoots and roots under 0°C. The data are means ± SE, *n* = 3. Different letters indicate a significant difference between means (*P* < 0.05).

The expression of several low temperature-related genes was also profiled to determine how their differences were influenced by rhizobium symbiosis in alfalfa. In most plants, *CBF2* functions as a cold-response transcriptional regulator. Despite differing in their rhizobium nodulation, when the plant groups were subjected to the 0°C-low temperature treatment they, similarly, expressed the *CBF2* gene. Nevertheless, the AN group had the highest transcript level for the *CBF2* gene in roots from 4 to 6 h, and after 8 h its transcription was significantly higher in the AN and IN groups than in plants without nodulation. In the shoots, the AN group displayed a higher transcript level of the *CBF2* gene earlier on, during 2–4 h of exposure to low temperature; yet after 6 h, its expression did not significantly differ among the three groups (Figure 6A,B).

Cas is a cold acclimation specific protein that, allows the plant to maintain functioning under a low temperature. In roots, rhizobium nodulation may help the host plant to maintain a higher level of *Cas15a* gene expression under a low temperature treatment. During the interval of 2–4 h, a significantly higher *Cas15a* gene expression was observed in the AN group compared with the IN and NN groups; hence, activated rhizobium symbiosis could have assisted host alfalfa plants’ respond to low temperature stress more rapidly. In the shoots, however, no significant difference was detected among the three groups during the first 4 h of low temperature, but later on, during the 6–8 h interval, the *Cas15a* gene expression in plants with AN was greater than those with IN or NN (Figure 6C,D).

As Figure 6E,F shows, *ProDH* gene expression in the shoots of the AN group was sustained at a constant level for the entire time period of the experiment, falling below that of the IN and NN groups after 6 h exposure to the 0°C-low temperature. Likewise, in the roots the expression of the *ProDH* gene remained constant and similar among the three groups from 0 to 6 h, but at 8 h the AN group had a lower transcript level of the *ProDH* gene.

**FIGURE 7:**
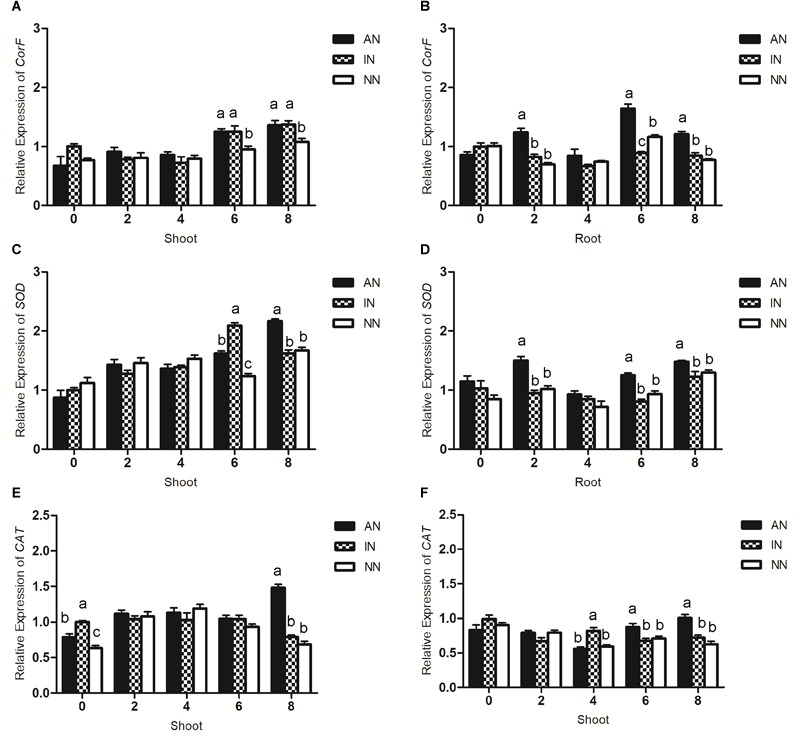
Effect of nodulation on the response of *CorF* **(A,B)**, *SOD* **(C,D)**, and *CAT* **(E,F)** genes to low temperature stress in alfalfa shoots and roots under 0°C. The data are means ± SE, *n* = 3. Different letters indicate a significant difference between means (*P* < 0.05).

The *CorF* gene contributes to soluble sugar accumulation. As shown in Figure 7A,B, nodulation in alfalfa significantly increased the expression of this gene in the shoots after 6 h of the 0°C-low temperature treatment. In the root part, compared with the other two groups, the AN group showed significantly higher *CorF* levels at 2, 6, and 8 h.

The expression levels of SOD and CAT are indicative of the antioxidative abilities of plants. The transcript level of SOD gradually increased in the shoot of the three groups during the first 4 h of the 0°C-low temperature treatment. At 6 h, both AN and IN groups had a significantly higher SOD levels when compared with the NN group. Moreover, an activated rhizobium induced a greater level of SOD in the AN group at 8 h. In roots, only the AN group showed higher SOD levels at 2, 6, and 8 h (Figure 7C,D).

When the low temperature treatment began, the IN group displayed a greater level of CAT than AN or NN did in the shoots of alfalfa. This difference, however, disappeared during the interval of 2–6 h. Through 8 h the AN group had the highest level of CAT in the shoots, whereas in the roots, although no such difference in CAT was observed in early 2 h, at 4 h it was significantly greater in the IN group than AN or NN, becoming highest in the AN group after 6 and 8 h (Figure 7E,F).

## Discussion

Low temperature is a major abiotic factor that limits the growth, development, survival, and productivity of plants (Zhou et al., 2018). Accumulating evidence shows that rhizobium symbiosis with plants plays an important role in their various abiotic stress tolerance mechanisms (Larrainzar and Wienkoop, 2017). In this experimental study, we demonstrated that rhizobium symbiosis could improve low-temperature tolerance in alfalfa. By inducing rhizobium nodules in its roots, the activity of anti-oxidation enzymes, osmotic adjustment, and low temperature-related genes of host plants were all significantly altered, consequently attenuating the oxidative stress, which led to higher survival under a low temperature condition.

Altered environmental factors may significantly change how symbionts behave when host plants must adjust to new circumstances (Paracer and Ahmadjian, 2000). In this study, two nutrient solutions were used to irrigate the alfalfa plants inoculated with or without rhizobia (Supplementary Figure S1). One nutrient solution provided plants with inorganic nitrogen, while another contained no nitrogen supply. Without nitrogen intake from nutrient solution, the symbiotic rhizobia were activated, forming the characteristic pink AN (AN group), which provide organic nitrogen in the form of amino acids to the host plant (Silvente et al., 2002). In contrast, plants irrigated with the nitrogen-containing nutrient solution could assimilate the inorganic nitrogen directly from the nutrient solution (Taule et al., 2012), leaving symbiotic rhizobia inactivated, thus generating the white IN (IN group). Compared with IN, more plants of the AN group survived under low temperature stress and exhibited stronger physiological responses, suggesting that an activated rhizobium nodule converted more nutrients for the host alfalfa to mitigate against the low temperature environment.

Both IN and NN groups were irrigated with the same total nitrogen nutrient solution, but though plants were inoculated with rhizobia the nodule was left inactive in the IN group, from which a higher proportion survived under low temperature stress. This result indicates that rhizobia symbiosis with the IN may have contributed to the low-temperature tolerance of host plants. This may due to rhizobia can enhance the systemic resistance of their host plants by inducing the expression of many defensive genes in different legume species, like Stylosanthes (*Stylosanthes. guianensis cv*. Reyan II) and peanut (*Arachis hypogaea* L.) (Dong et al., 2017; Furlan et al., 2017).

Alfalfa, moreover, is an autotetraploid legume crop (Brouwer et al., 2000). As such, there may be much natural variation in different growth and physiological parameters between different individual plants of the same cultivar. Therefore, for our experiment, a great many plants were needed to carry out a robust statistical analysis, with a total of 4500 (2250 plants for each temperature × 2 temperature) alfalfa individuals used to quantify survival.

To explore the mechanisms underpinning plant responses to low temperature stress, we investigated key physiological variables responsible for low-temperature tolerance. Zhang et al. (2011) had determined the survivorship of *Medicago truncatula* cv. Jemalong A17 and *Medicago falcata* cv. Humeng at -10°C for 5 h. By contrast, in our study we ensured that aboveground and belowground plant tissues experienced the same low temperature. Under –6°C, alfalfa with AN survived best, which indicated the rhizobium interaction improved this plant’s low-temperature tolerance.

It has been reported that low temperatures induce cell membrane damage (Aghdam et al., 2019), and MDA is widely adopted as an indicator of oxidative stress and membrane integrity in plants when they respond to stressors (Sato et al., 2011). In our study, under a low temperature, compared with both IN and NN groups, the AN group clearly produced less MDA in both their shoot and root parts (Figure 3). Recently, a similar difference in the accumulation of MDA was reported between two genotypes of bermudagrass [*Cynodon dactylon* (L). Pers.] that differed in their tolerance to low temperature stress (Huang et al., 2017). Undoubtedly, higher plants have developed many complex strategies to respond to the low temperature-induced stress, and research has suggested that plants exhibit higher POD and SOD under conditions of low temperature stress, and then benefit from receiving less oxidative stress (Radwan et al., 2010). Furthermore, enhanced POD activity can indicate a higher capacity for the decomposition of H_2_O_2_ that is generated by SOD (Wu et al., 2014). Our results revealed that among the three alfalfa groups, POD (in root) and SOD activity was greatest in the AN group after 8 h at 0°C (Figure 4B–D), and we also found the IN group’s POD activity in shoots exceeded that of NN (Figure 4A), which together suggested POD accumulation is a key factor promoting alfalfa’s survival under low temperature. Furthermore, the findings point to an inactivated nodule facilitating higher cold tolerance for host plants. This may indicate that rhizosphere form composition was changed after the rhizobium inoculation, so that the “stress tolerance ability” for plant was increased. Concurrently, there was markedly higher SOD activity in the roots with AN than those with IN or NN (Figure 4D), indicating that alfalfa plants with functional nodules had a stronger tolerance to low temperature. Further, since the AN group received less oxidative stress, this may be due to the fact that rhizobium symbiosis stimulates host plants to produce additional antioxidants. Our results are consistent with those of Zhang R.X. et al. (2017) and Kakar et al. (2016), who found that cold-resistant plants received less oxidative stress and were capable of higher antioxidant enzyme activity. It is known that antioxidant metabolism can protect cells from oxidative damage caused by ROS, and that CAT can decrease oxidative damage (Meng et al., 2017). The lower MDA accumulation in the AN group may explain, in part, its milder oxidative damage in AN group which also has higher activity of CAT. The greater up-regulation of SOD and CAT biosynthesis genes in the AN group corroborates the higher activity of both antioxidant enzymes we found. Our study is in line with view taken by Mutlu et al. (2013) opinion, who pointed out that cold-tolerant plant cultivar should have higher CAT activity when faced with cold stress. To sum up, the more MDA accumulated and lower antioxidant enzyme activity in IN and NN groups indicated that those plants suffered more severe oxidative damage under low temperature stress. However, with the aid of its rhizobia symbionts the AN group incurred less damage.

Through a variety of metabolic pathways, the plant cell releases soluble organic matter or compounds to reduce its water potential and to adjust itself to the surrounding stressful environment (Cunningham et al., 2003). We also determined the accumulation of soluble substances such as proline, soluble sugar, and soluble protein in alfalfa plants. High-level accumulations of soluble substance should enhance a plant’s freezing tolerance (Cao et al., 2012), and proline can also function as a protein compatible hydrotrope (Srinivas and Balasubramanian, 1995), which may positively affect soluble proteins and promote the latter to accumulate. It was reported that cold treatment could increase accumulation of soluble protein (Castonguay et al., 1995). Bao et al. (2017) had pointed out that an increased protein content of alfalfa (*M. sativa cv.* Dongmu) prevents damage from cold stress, and soluble sugar accumulation was associated with enhanced freezing tolerance in curly kale (*Brassica oleracea* L. var *acephala*; Steindal et al., 2015). Compared with the IN group, AN can obtain more soluble substances from the active rhizobia symbiotic nodules to counteract an environmental stress (Erdal, 2012). After 8 h of low temperature stress, the AN group had accumulated more proline than the IN and NN groups, and similar results were found for soluble protein and sugar. This clearly shows that the plant-nodule interaction is vital for improving the low-temperature tolerance of alfalfa.

Numerous molecular processes were also altered when the alfalfa plants faced low temperature stress. According to Ito et al. (2006), the *CBF* gene could be critical for plants’ cold tolerance, which when overexpressed can improve plant cold tolerance. Furthermore, the study pointed out the accumulation of proline and soluble sugar in rice under abiotic stress could have been due to the overexpression of its *CBF* gene (Ito et al., 2006). Similarly, our results showed that the AN group expressing more *CBF* genes also accumulated more proline and soluble sugar than IN and NN groups. Upregulated expression of the *Cas* gene in AN may have led to more dehydrins which protected the structure of cells and maintained the stability of intracellular proteins as well as the activity of intracellular macromolecules, thereby enhancing the low-temperature tolerance of host plants (Monroy et al., 1993; Pennycooke et al., 2008; Hara, 2010). Conversely, Cas may regulate the process of proline synthesis in addition to sugar content (Zuther et al., 2015). Under low temperature, elevated proline may protect the stressed plant from dehydration and stabilize its subcellular structure and, more importantly, scavenge for free radicals (Ashraf and Foolad, 2007). In short, proline accumulation fosters a low-temperature tolerance of host plants. Nevertheless, proline dehydrogenase (ProDH), a key enzyme in proline degradation, constitutively consumes extra proline in plant cells. In our study, alfalfa plants subjected to a low temperature treatment responded with more *ProDH* gene expression that led to a reduced proline concentration, however, rhizobium inoculation was able to change this gene’s expression profile. During the low temperature treatment, transcription of the *ProDH* gene was sustained at a constant level in shoots of the AN group, and even decreased in its roots (Figure 5A,B, 6E,F). It has been suggested that *ProDH* is a gene expressed in all plant tissues in model plant (Liu et al., 2012), thus, the effect of rhizobium on host plants may first act in the roots and then transferred to the aboveground parts. Moreover, CorF is a key enzyme in the formation of raffinose family oligosaccharides (RFOs) (Pembleton and Sathish, 2014). Work by Cunningham et al. (2003) indicated RFO synthesis strengthens the overwintering ability of plants. In our study, compared with IN and NN, the AN group had higher transcript levels of *CorF* gene. This may partially explain why shoots, as well as roots, of the AN group also showed a higher soluble sugar content. We speculate the changed gene expression induced by low temperature stress may be regulated by the symbiotic relationship between rhizobia and its host plants.

In a nutshell, based on our study’s results, we conclude that rhizobium inoculation effectively protected the alfalfa’s membrane system and assisted host plants to accumulate more proline, soluble protein, and soluble sugar; induced cold stress-related genes to counter the low temperature stress; and activated the interaction between nodules and alfalfa plants, which together provided a better protective effect against low temperature stress.

## Author Contributions

T-MH and P-ZY conceived and designed the project. Y-SL performed the experiments, analyzed the data, and wrote the manuscript. J-CG, X-YS, and Y-XZ performed the experiments. All authors contributed to the manuscript revision, and read and approved the submitted version.

## Conflict of Interest Statement

The authors declare that the research was conducted in the absence of any commercial or financial relationships that could be construed as a potential conflict of interest.

## Abbreviations

Cas: the cold acclimation specific protein
CAT: catalase
*CBF2*: a transcription factor related to the cold tolerance
*CorF*: the coding gene for galactinol synthase
MDA: malondialdehyde
POD: peroxidase
*ProDH*: the coding gene for proline dehydrogenase
qRT-PCR: quantitative real-time PCR
ROS: reactive oxygen species
SOD: superoxide dismutase
