# Supplementary material for: Effect of Rhizobium Symbiosis on Low-Temperature Tolerance and Antioxidant Response in Alfalfa (Medicago sativa L.)
Source: Front Plant Sci. 2019 Apr 30;10:538. doi: 10.3389/fpls.2019.00538 (PMC6503086; doi:10.3389/fpls.2019.00538)
Supplement: Supplementary file 1 [file Table_1.DOCX]

Supplementary Material

Effect of rhizobium symbiosis on low temperature tolerance and antioxidant response of alfalfa

Yu-shi Liu, Jin-Cai Geng, Xu-Yang Sha, Yi-Xin Zhao, Pei-Zhi Yang^*^, Tian-Ming Hu*

*** Correspondence:** Peizhi Yang [yangpeizhi@126.com](mailto:yangpeizhi@126.com)

Tianming Hu [hutianming@126.com](mailto:hutianming@126.com)

# Supplementary Figure


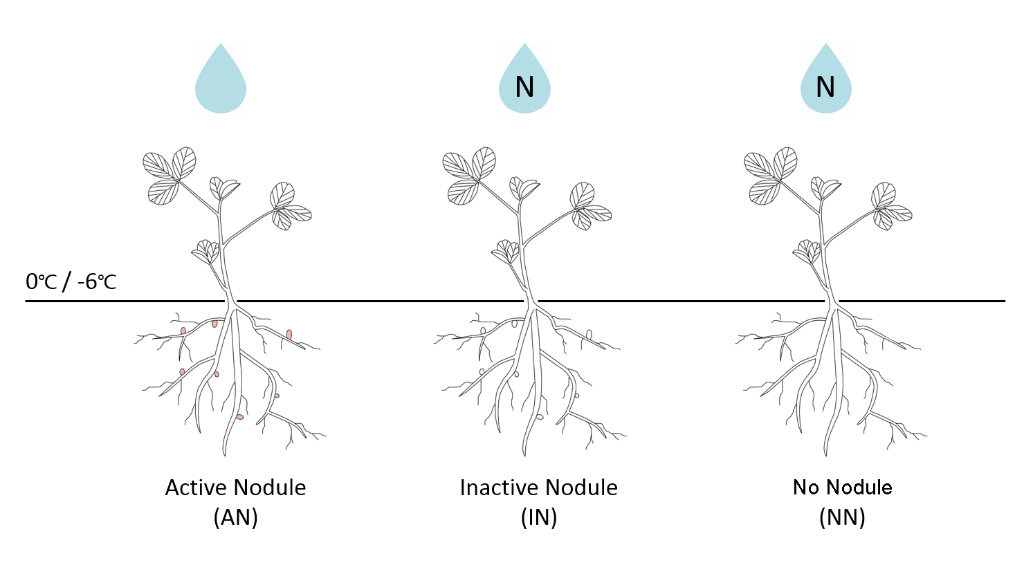


**Supplementary Figure S1.** Schematic illustration of experimental design of this study.


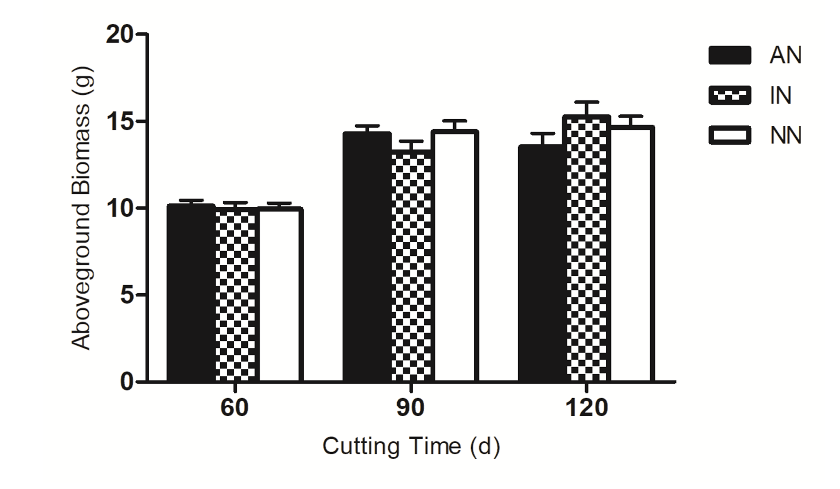


**Supplementary Fig S2.** The aboveground biomass of cutting (The data are means ± SE. n=3. Different *letters* indicate significant difference *P* < 0.05.)


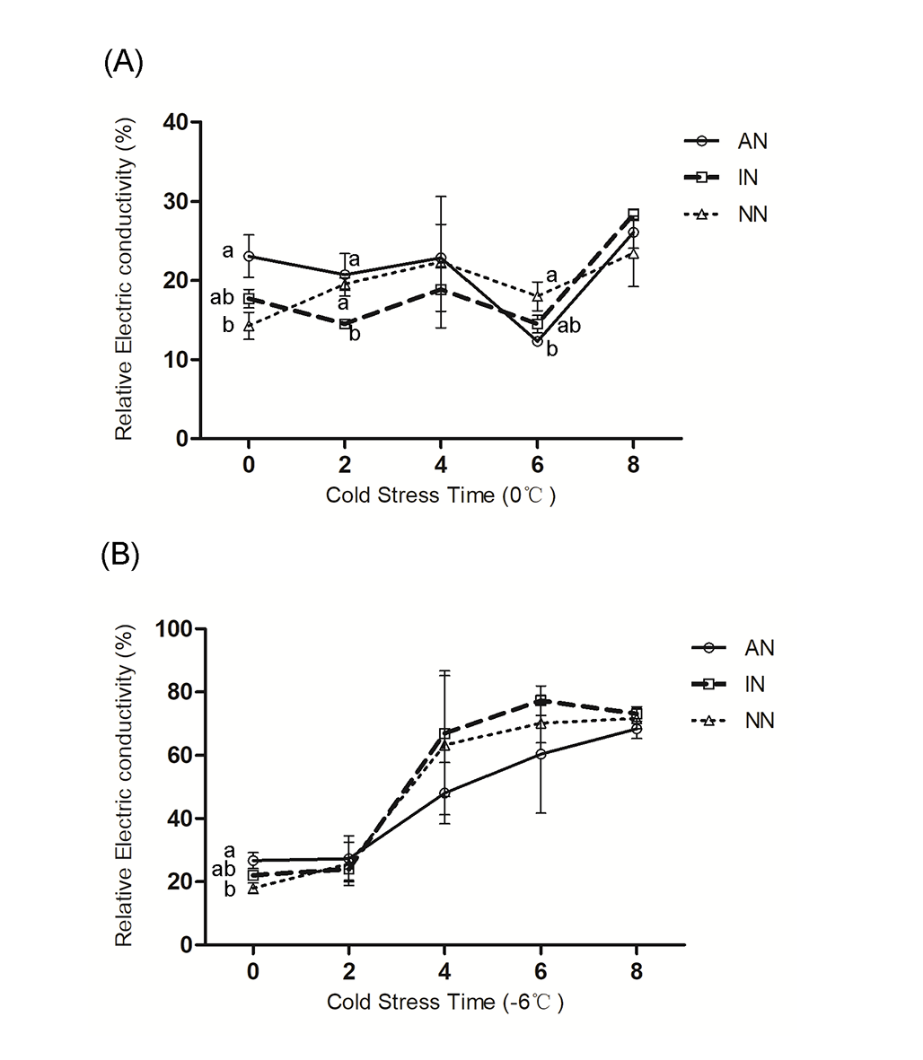


**Supplementary Figure S3.** Effect of relative electrical conductivity in alfalfa leaf. **A** Under 0℃; **B** Under -6℃. (The data are means ± SE. n=3. Different *letters* indicate significant difference *P* < 0.05.)

# Supplementary Tables

# Supplementary Table S1. Primer sequences used for real-time quantitative RT-PCR

| **GenBank accession number** | **Gene** | **Primer sequence (5 ′-3 ′)** |
| --- | --- | --- |
| JQ028730 | *β-Actin* | TTTGAGACTTTCAATGTGCCCGCC |
|  |  | TAGCATGTGGGAGTGCATAACCCT |
| L12461 | *Cas15* | GAAGACCACAAGGAGGGATTT |
|  |  | GTTCATGACCCTCTCCATGTT |
| EU139867 | *CBF2* | CTGCGACGACTCCAAAGAA |
|  |  | CCTCATTTCACACACCCATTTATC |
| AY556386 | *ProDH* | GCTGAGATGGCAGAAGAAAGA |
|  |  | GCTCTGGTCTCTTCCTTGTATG |
| AY126615 | *CorF* | CCAACATCACCAACACTCAATC |
|  |  | CCAACAACACCCTTCACATAATC |
| XM_013606079 | *SOD* | CCAGGACCCATTGGTTACTAAA |
|  |  | CTGGTCTGACGTTCTTGTACTG |
| XM_013606823 | *CAT* | GAAGTGTCTGTTGGAGGAAGAG |
|  |  | CAGGATAGTTACCAGCAGCAA |

**Supplementary Table S2** Statistical results of the one-way analysis of variance on the effect of the nodulation on alfalfa aboveground biomass.

| Factor | Aboveground Biomass | |
| --- | --- | --- |
|  | *F* | *P* |
| 60d | 0.100 | 0.905 |
| 90d | 1.291 | 0.284 |
| 120d | 1.330 | 0.273 |

**Supplementary Table S3** Statistical results of the one-way analysis of variance on the effect of the cold stress time on alfalfa leaf relative electric conductivity.

| Factor | | Leaf Relative Electric Conductivity | | | |
| --- | --- | --- | --- | --- | --- |
|  |  | 0℃ | | -6℃ | |
|  |  | *F* | *P* | *F* | *P* |
| Stress time | 0 | 5.403 | 0.012 | 6.337 | 0.006 |
|  | 2 | 7.792 | 0.002 | 0.176 | 0.840 |
|  | 4 | 0.199 | 0.821 | 0.522 | 0.600 |
|  | 6 | 5.159 | 0.014 | 0.758 | 0.480 |
|  | 8 | 2.197 | 0.133 | 0.441 | 0.648 |

**Supplementary Table S4** Statistical results of the one-way analysis of variance on the effect of the cold stress time on alfalfa survival rate and the content of MDA.

|  | Factor | | Survival Rate | | MDA | |
| --- | --- | --- | --- | --- | --- | --- |
|  |  |  | *F* | *P* | *F* | *P* |
| Shoot | Stress time | 0 | - | - | 0.707 | 0.503 |
|  |  | 2 | - | - | 2.094 | 0.145 |
|  |  | 4 | 0.086 | 0.919 | 18.568 | ＜0.001 |
|  |  | 6 | 25.229 | 0.001 | 3.925 | 0.034 |
|  |  | 8 | 12.000 | 0.008 | 9.282 | 0.001 |
| Root | Stress time | 0 |  |  | 0.928 | 0.409 |
|  |  | 2 |  |  | 0.174 | 0.841 |
|  |  | 4 |  |  | 4.411 | 0.023 |
|  |  | 6 |  |  | 7.647 | 0.003 |
|  |  | 8 |  |  | 51.367 | ＜0.001 |

**Supplementary Table S5** Statistical results of the one-way analysis of variance on the effect of the cold stress time on the activities of POD, SOD and CAT of alfalfa.

|  | Factor | | POD | | SOD | | CAT | |
| --- | --- | --- | --- | --- | --- | --- | --- | --- |
|  |  |  | *F* | *P* | *F* | *P* | *F* | *P* |
| Shoot | Stress time | 0 | 0.194 | 0.825 | 4.686 | 0.019 | 11.959 | ＜0.001 |
|  |  | 2 | 87.592 | ＜0.001 | 0.616 | 0.546 | 28.370 | ＜0.001 |
|  |  | 4 | 3.992 | 0.032 | 1.878 | 0.175 | 8.992 | 0.001 |
|  |  | 6 | 13.187 | ＜0.001 | 1.063 | 0.361 | 0.833 | 0.447 |
|  |  | 8 | 0.449 | 0.643 | 3.541 | 0.045 | 6.315 | 0.006 |
| Root | Stress time | 0 | 5.796 | 0.009 | 0.297 | 0.746 | 1.153 | 0.333 |
|  |  | 2 | 9.142 | 0.001 | 11.840 | ＜0.001 | 1.802 | 0.187 |
|  |  | 4 | 1.125 | 0.341 | 14.445 | ＜0.001 | 0.079 | 0.925 |
|  |  | 6 | 1.595 | 0.224 | 6.785 | 0.005 | 4.024 | 0.031 |
|  |  | 8 | 6.473 | 0.006 | 5.741 | 0.009 | 1.231 | 0.310 |

**Supplementary Table S6** Statistical results of the one-way analysis of variance on the effect of the cold stress time on the content of proline, soluble protein and soluble sugar of alfalfa.

|  | Factor | | Proline | | Soluble protein | | Soluble sugar | |
| --- | --- | --- | --- | --- | --- | --- | --- | --- |
|  |  |  | *F* | *P* | *F* | *P* | *F* | *P* |
| Shoot | Stress time | 0 | 7.314 | 0.003 | 4.626 | 0.020 | 13.005 | ＜0.001 |
|  |  | 2 | 6.389 | 0.006 | 7.429 | 0.003 | 0.554 | 0.582 |
|  |  | 4 | 8.554 | 0.002 | 27.584 | ＜0.001 | 7.285 | 0.003 |
|  |  | 6 | 4.484 | 0.022 | 6.433 | 0.006 | 18.364 | ＜0.001 |
|  |  | 8 | 1.936 | 0.166 | 40.830 | ＜0.001 | 3.407 | 0.049 |
| Root | Stress time | 0 | 7.355 | 0.003 | 2.032 | 0.153 | 10.805 | ＜0.001 |
|  |  | 2 | 0.261 | 0.773 | 6.290 | 0.006 | 28.495 | ＜0.001 |
|  |  | 4 | 3.196 | 0.059 | 3.195 | 0.059 | 45.392 | ＜0.001 |
|  |  | 6 | 16.448 | ＜0.001 | 3.843 | 0.036 | 1.216 | 0.314 |
|  |  | 8 | 5.435 | 0.011 | 30.109 | ＜0.001 | 22.048 | ＜0.001 |

**Supplementary Table S7** Statistical results of the one-way analysis of variance on the effect of the cold stress time on the relative expression of *CBF2*, *Cas15*, *ProDH* genes in alfalfa.

|  | Factor | | *CBF2* | | *Cas15* | | *ProDH* | |
| --- | --- | --- | --- | --- | --- | --- | --- | --- |
|  |  |  | *F* | *P* | *F* | *P* | *F* | *P* |
| Shoot | Stress time | 0 | 0.559 | 0.579 | 2.938 | 0.072 | 2.414 | 0.111 |
|  |  | 2 | 17.702 | ＜0.001 | 1.129 | 0.340 | 0.790 | 0.465 |
|  |  | 4 | 158.430 | ＜0.001 | 0.296 | 0.747 | 3.017 | 0.068 |
|  |  | 6 | 2.694 | 0.088 | 74.224 | ＜0.001 | 8.737 | 0.001 |
|  |  | 8 | 1.085 | 0.354 | 83.391 | ＜0.001 | 11.734 | ＜0.001 |
| Root | Stress time | 0 | 0.650 | 0.531 | 24.091 | ＜0.001 | 2.150 | 0.138 |
|  |  | 2 | 4.419 | 0.023 | 127.369 | ＜0.001 | 0.401 | 0.674 |
|  |  | 4 | 8.406 | 0.002 | 14.327 | ＜0.001 | 0.066 | 0.936 |
|  |  | 6 | 14.516 | ＜0.001 | 5.933 | 0.008 | 1.819 | 0.184 |
|  |  | 8 | 34.156 | ＜0.001 | 3.114 | 0.063 | 36.999 | ＜0.001 |

**Table S8** Statistical results of the one-way analysis of variance on the effect of the cold stress time on the relative expression of *CorF*, *SOD*, *CAT* genes in alfalfa.

|  | Factor | | *CorF* | | *SOD* | | *CAT* | |
| --- | --- | --- | --- | --- | --- | --- | --- | --- |
|  |  |  | *F* | *P* | *F* | *P* | *F* | *P* |
| Shoot | Stress time | 0 | 3.120 | 0.062 | 1.794 | 0.188 | 26.952 | ＜0.001 |
|  |  | 2 | 0.970 | 0.393 | 0.362 | 0.240 | 0.441 | 0.649 |
|  |  | 4 | 0.795 | 0.463 | 2.583 | 0.096 | 1.075 | 0.357 |
|  |  | 6 | 5.534 | 0.011 | 88.464 | ＜0.001 | 1.768 | 0.192 |
|  |  | 8 | 6.114 | 0.007 | 38.673 | ＜0.001 | 107.299 | ＜0.001 |
| Root | Stress time | 0 | 2.544 | 0.100 | 2.372 | 0.115 | 2.092 | 0.145 |
|  |  | 2 | 34.775 | ＜0.001 | 26.826 | ＜0.001 | 3.251 | 0.056 |
|  |  | 4 | 1.710 | 0.202 | 2.224 | 0.130 | 17.861 | ＜0.001 |
|  |  | 6 | 59.825 | ＜0.001 | 26.233 | ＜0.001 | 6.711 | 0.0049 |
|  |  | 8 | 33.668 | ＜0.001 | 5.100 | 0.014 | 21.617 | ＜0.001 |
